# Integrating Real-World Evidence in the Regulatory Decision-Making Process: A Systematic Analysis of Experiences in the US, EU, and China Using a Logic Model

**DOI:** 10.3389/fmed.2021.669509

**Published:** 2021-05-31

**Authors:** Meng Li, Shengqi Chen, Yunfeng Lai, Zuanji Liang, Jiaqi Wang, Junnan Shi, Haojie Lin, Dongning Yao, Hao Hu, Carolina Oi Lam Ung

**Affiliations:** State Key Laboratory in Quality Research of Chinese Medicine, Institute of Chinese Medical Sciences, University of Macau, Macao, China

**Keywords:** regulatory science, real-world evidence, decision-making, logic model, regulation

## Abstract

Real world evidence (RWE) and real-world data (RWD) are drawing ever-increasing attention in the pharmaceutical industry and drug regulatory authorities (DRAs) all over the world due to their paramount role in supporting drug development and regulatory decision making. However, there is little systematic documentary analysis about how RWE was integrated for the use by the DRAs in evaluating new treatment approaches and monitoring post-market safety. This study aimed to analyze and discuss the integration of RWE into regulatory decision-making process from the perspective of DRAs. Different development strategies to develop and adopt RWE by the DRAs in the US, Europe, and China were reviewed and compared, and the challenges encountered were discussed. It was found that different strategies on development of RWE were applied by FDA, EMA, and NMPA. The extent to which RWE was adopted in China was relatively limited compared to that in the US and EU, which was highly related to the national pharmaceutical environment and development stages. A better understanding of the overall goals, inputs, activities, outputs, and outcomes in developing RWE will help inform actions to harness RWD and leverage RWE for better health care decisions.

## Introduction

Real-World Data (RWD) refers to the data obtained through multiple sources, which is related to patient health status or delivery of health care and medical behavior in routine clinical practice (1, 2). Data sources include electronic health records (EHR), medical claim data, medical databases and patient information collected from various devices. Real-World Evidence (RWE) denotes the analysis of clinical research evidence generated by RWD related to the use of medical products and potential benefits or risks. The definitions and applications of RWE are closely linked not only to the development of national healthcare policy but also to the regulatory science (3). Traditionally, regulatory approvals of new drugs have always been largely, if not solely, based on randomized clinical trials (RCTs). The rigor of RCT study design mandates a set of eligibility criteria for subject inclusion and exclusion to ensure homogeneity and representativeness of the studying findings (4). Many RCTs exclude patients who have multiple comorbidities to ensure the internal validity of the findings. To certain extent, the representation of people with multiple comorbid conditions can be easily compromised with the exclusion of population subsets in RCTs. Inevitably, important information for developing the evidence base about the proper use of a treatment or intervention for population subsets that RCTs are able to generate is limited.

Compared with RCTs, RWE plays an important complementary role to RCTs with much needed information from real-life practices during the life cycle evidence of drugs to support regulatory decision-making. The guideline of updated international draft of the “General considerations for clinical studies, ICH E8 (R1)” states that RWE generated by pragmatic trial that embeds randomization within EHR and claims data could provide insight into post-marked safety issues, inform clinical care practices and avoid adverse events.^1^ In terms of the development of treatment interventions for rare disease, RCT designs are difficult to recruit enough patient populations (5). Evidence-based clinical practice for drug development and approval should incorporate more data sources, like EHR claim data, social media data and large volume of data created by medical devices. So RWE could provide opportunities and be complementary to RCTs. At present, RWE is being increasingly used to inform regulatory decisions. Differences in the definition, constitution, scope, and applications of RWE among countries contribute to the diversity of the RWE regulations (6).

The majority of the current literature on RWE focuses on how to effectively use RWE in supporting drug clinical development and evaluation, assisting drug regulatory decision-making (e.g., pharmacovigilance and post-marketing research) (7), evaluating clinical treatment effects and how to effectively derive RWE from RWD (e.g., quality control and risk of bias assessment) (8). Less attention had been paid to the organizational perspective about how to develop, adopt, and advance RWD, and how such an approach might be generalized across different regulatory settings to benefit the efficiency of regulatory actions. In particular, there was little systematic analysis about how RWE can be adopted by the drug regulatory authorities (DRAs). The DRAs in the United States (US) and Europe Union (EU) have accumulated rich practical experience in using RWD to evaluate the safety of medical products in the past decades (9). Notably, the development of RWE in China has also been significantly improved in the past few years, which was originally used to evaluate the outcomes and comparative effectiveness of the traditional Chinese medicine interventions (10). Therefore, the use of RWE in DRAs of these three countries/regions was analyzed and compared in order to describe the current development and application status in the context of three representative health care systems. Thus, this study aims to analyze and discuss the integration of RWE into regulatory decision-making process from the perspective of DRAs in the US, EU and China. The main objectives of the study are to: (1) systematically collect RWD/RWE related information from different sources including the three DRAs' official websites and academic databases; (2) analyze how the RWD/RWE was generated and applied in the US, EU, and China based on the logic model; and (3) explore the current development, applications and implications of RWD/RWE in the US, EU, and China. It is envisioned that this study findings would be able to help inform actions to harness RWD and leverage RWE for better health care decisions.

## Methods

### Research Design and Data Collection

#### Data Mining

Search of national medical regulatory agencies in the US, EU, and China was carried out, and the corresponding datasets were generated. Afterwards, documentary analysis was adopted in this study. Firstly, textual information of the policy and application of RWE in the US, EU, and China was collected from the corresponding official websites and databases. Inclusion criteria were designed based on specific institutional settings as summarized in Table 1. To minimize the possibility of missing relevant information, the search term used in this study was “real world.” The detailed retrieval process was as follows:

**Table 1:** Variables and inclusion criteria in the documentary analysis.

**Variables**	**Inclusion criteria**
Country/region	United States, European Union, and China
Language	English or Chinese
Main content	Regulatory guidelines, proposals, or reports of initiatives/promotion actions and media reports
Scope of documentation	Medicines, biologics, advanced therapy medicinal products, herbal medicine/traditional Chinese medicine, and medical devices for human use
Issuing time	Documents released between 2010 (January) and 2021(March)

- US: The term “real world” was used in the search at the “Search for FDA guidance documents” function on the U.S. Food and Drug Administration's (FDA) official website.^2^ In addition to the FDA website, the same term was also searched in the official website of the Federal Register, then the documents released from FDA were kept.^3^ The Federal Register is the federal government gazette, which mainly include federal laws, government agency rules, proposed rules, project descriptions and public notices.
- EU: The term “real world” was used in the search at the official website of the European Medicines Agency (EMA).^4^ “News” and “Events” were chosen to yield the types of information relevant to this study. All options in the categories were selected except “Veterinary.” Similarly, the same term was also used to search in the official website of the European Commission-legislation.^5^ The documents issued by “European Commission,” “The council of the EU,” “European parliament,” and “European Council” were kept for further analysis. These four institutions are the central legislative bodies of the EU, which play the most important role in EU decision-making (11).
- China: The term “real world” was searched in the “Regulatory Documents” and “Government Affairs” sections on the official website of the National Medical Products Administration (NMPA) in China.^6^ More information about the initiatives was selected from the website of “China Pharmaceutical Information” by searching the same term.^7^ This website is constructed by Information Center of NMPA, which provides food and medicine supervision policies, regulations, and pharmaceutical industry information in China since 1996.

#### Data Screening and Analysis

Manual data screening was conducted by selecting all documents containing or related to “real world data” or “real world evidence.” This process was conducted by two independent researchers. (1) Each researcher first screened individually, (2) The two researchers cross-checked each other's search results and any inconsistent results were discussed, and (3) upon discussion, the consistent and agreed (inconsistent results) documents were included for further analysis. Subsequently, the logical model was used to analyze the data from the three DRAs. In particular, logic model is a schematic depiction that presents the process of how an intervention produces its outcomes. It is usually used to help stakeholders to consider and design the interventions, which may lead to different outcomes and impacts. It has been applied in this research to analyze and guide the planning, description, execution, management, and evaluation of a policy or a strategy (12). Developing strategies could be optimized by the performance of each aspect and the overview of the entire existing program. A logic model may serve one or more purposes/aims, which imply the motivations of the program. It normally comprises of four main components: input (resources), activities, outputs, and outcomes, which are determined based on a set of predefined aims or objectives of the program of interest (13). Inputs refer to the resources to be available for the program, such as financial support, personnel and technical assistance. Activities are the events or actions essential to produce desired outcomes. Outputs refer to the direct results of the activities like regulatory guidelines or any communities and platforms. Outcomes refer to the results that the program is set to achieve eventually.

All regulatory documents, initiatives proposals or media reports were classified and analyzed following the framework of logic model after data screening. In this study, the aims of developing and adopting RWE were extracted from the requirements of the national government/congress/parliament for the development of RWE. Input and activities were merged in this study, including the resource input, and projects/workshops operation. Outputs were the direct effects produced by the input and activities, such as published reports and articles, issued policy guidelines, established alliances/databases, etc. Outcome referred to the promotion of medicine or the impact on society due to the development of RWE, such as the review and approval of presentative medicines and devices, and the reference role for other regulatory decision-making.

## Results

Four regulatory documents related to “real world” were retained on the FDA official website, and 105 related documents were obtained from the Federal Register at data mining stage. Similarly, 263 and 94 results were returned from the EMA and EC legislation websites, respectively. In China, a total of 34 regulation documents and government affairs were found on the NMPA official website, and there were 17 related information on the China Pharmaceutical information website. Data irrelevant to RWD/RWE and the duplication were removed in the manual screening stage. There were 31, 57, and 34 documents/reports remained in the US, EU, and China for follow-up analysis. It was noted that each document/report may contain different elements of the logic model framework. The comparative analysis results were summarized as bellow.

### United States—Food and Drug Administration

#### The Aims of FDA for RWE Development

There are two main documents issued by the U.S. Congress to regulate FDA to develop RWE in different fields based on specific aims. The first one was the 21st Century Cures Act (14), which was announced on December 13, 2016, which decided to add 505F (21 U.S. Code 355 g.) “Utilizing Real World Evidence” in the Federal Food, Drug, and Cosmetic Act. RWE was defined as “data regarding the usage, or the potential benefits or risks, of a drug derived from sources other than traditional clinical trials.” Congress required FDA to establish an implementation framework based on this section within 2 years to assess potential utilizing of RWE, including supporting the approval of new indications for approved drugs and post-approval research requirements. Collaborations of FDA with regulated industry, academia, and medical professional organizations should be carried out in specific requirements. At the same time, Congress required FDA to implement this framework no later than 3 years and to issue a draft guidance for industry within 5 years.

Prescription Drug User Fee Amendments (PDUFA VI) year 2018–2022 was another document issued by Congress,^8^ which focused on speeding up and refining the drug review process in FDA. This edition stated that by the end of fiscal 2018, the FDA would complete one or more public seminars to gather opinions on the use of RWE in regulatory decision making. At the end of fiscal year 2019, FDA will fund appropriate activities to address key considerations when using RWE to make regulatory decisions, including pilot studies or methodological development projects. At the end of fiscal 2021, FDA will draft industry guidance on using RWE to evaluate safety and effectiveness in regulatory submissions, such as approval requirements and post-approval commitments for new indications.

### Inputs and Activities

#### Knowledge and Technology Support

FDA provided professional training for staff. FDA established an internal website in December 2017 for FDA staff to participate in supporting FDA's activities in evaluating RWE and its use in regulatory decisions. In 2019, Jacqueline Corrigan-Curay, the director of Medical Policy Office of FDA's Center for Drug Evaluation and Research (CDER), stated in a public report that efforts should be increased to enhance the internal education of FDA staff in RWD and RWE, including continuing to hold public meetings to get more expert opinions and to make standards for assessing RWD and RWE.^9^ In addition to training, FDA established the RWE Subcommittee of CDER's Medical Policy and Program Review Council, where the subcommittee aimed to assist FDA centers in evaluating RWE and propose advice for policy development. Specifically, the staff of the Office of New Drugs could consult the RWE Subcommittee in assessing the use of RWD/RWE to support regulatory decisions. This subcommittee provided an interactive platform on how to use RWE in promoting decision making and meet the requirements of Congress for the development of RWD/RWE.

#### Research and Project Funding

FDA cooperated with and funded various institutes to jointly promote and develop the use of RWD/RWE. In September 2009, the FDA signed a 4-year, $72 million contract with Harvard Pilgrim Healthcare institute. This project aimed to establish the “Mini-Sentinel Coordination Center” (15), which lay the foundation for the full implementation of the sentinel system. FDA also cooperated with some academic institutions and US government institutions, including projects, workshops or events. For example, the Duke-Margolis Center had been a key partner of the FDA. Duke-Margolis Center for Health Policy RWE Collaborative at Duke University was established in 2018. This collaborative effort aimed to inform the development of guidance, polices around use of RWD and RWE in decision making and had published several white papers. A series of workshops and meetings aiming to engage various stakeholders to improve the development of RWE were hosted. The priority areas were RWE endpoints roadmap, external comparators, shared real-world evidentiary opportunities, and etc. The applications of RWE in post-COVID-19 environment was also discussed in late 2020.^10^ FDA also co-hosted some events and seminars with Clinical Trial Transformation Initiative (16), University of Maryland Center of Excellence in Regulatory Science and Innovation (2), and American Association for Cancer Research^11^ on the topics related the use the RWE, including how to evaluate RWE generated by RWD in randomized trials, how to use the evidence generated by medical devices in the real world to improve device safety and real world clinical research.^12^ FDA conducted RWE related collaborative projects with University of California, San Francisco (UCSF)—Stanford Center of Excellence in Regulatory Science and Innovation (UCSF-Stanford CERSI). Harvard Pilgrim Health Care Institute's Department of Population Medicine (DPM) and Brigham and Women's Hospital (BWH)/Harvard Medical School also have RWE related projects with FDA. In addition to the workshops and projects, FDA's Office of Blood and Oncology Products had partnered with HHS IDEA Lab to co-sponsor the “Information Exchange and Data Transformation (INFORMED) Initiative” to establish an organization for technology and big data analysis infrastructure (17).

#### Workshops and Projects

A series of relevant RWD/RWE workshops and projects were organized by FDA and academic/government institutions since 2016. The main topics focused on: (1) the use and regulatory acceptability of RWD and RWE; (2) enhancing the use of RWD to generate RWE in regulatory decision-making; (3) the use of RWD to plan eligibility criteria and enhance recruitment; (4) leveraging RCT to generate RWE for regulatory purposes; and (5) building the national evaluation system for medical devices. Different demonstration projects produced several research publications/reports, frameworks, platforms, and research centers or organizations. For instance, OneSource is the platforms developed by FDA and UCSF-Stanford CERSI for collecting clinical trial data and design specific methods for transmitting health information to test and shape data, methods and analytic standards for drug development and RWE utilization. One demonstration project of FDA-Catalyst is the open source FDA-My Studies APP (18), which is a new mobile technology to gather RWE from patient mobile devices. RCT Duplicated demonstration project was launched with FDA and Brigham and Women's Hospital in 2017, which develops substantial assessment of the comparability of randomized and non-randomized designs to understand if non-interventional designs could provide credible evidence of drug effect. This project also explores the possibility of using RWE to replicate the results of RCTs in order to predict the results and findings of ongoing phase IV trials.^13^

In 2020, the COVID-19 pandemic has brought urgent medical and public health challenges worldwide. Related online workshops and meetings were also hosted by FDA to discuss the opportunities of using RWE to respond to this pandemic (e.g., evaluating the potential therapies and diagnostics) and how to use RWE to assess the effectiveness of preventive vaccines. FDA has promoted the use of RWD for the COVID-19 pandemic, such as using RWD to understand diseases, plan clinical trials, and manage medical product supply chains to prevent shortages. RWD has potentials to help identify, evaluate, and provide an initial understanding of the characters of COVID-19 diagnostics and effectiveness of therapies. FDA partnered with the Reagan-Udall Foundation and Friends of Cancer Research launched an initiative named COVID-19 Evidence Accelerator where natural history of the disease, epidemiology information, and clinical outcomes, like mortality, hospitalization, and the number of intensive care units were collected to answer questions about COVID-19.

### Outputs

#### Publications and Reports

From Web of Science database, the academic outputs regarding “RWD/RWE” from FDA were summarized. Fifty-four records were generated with the top document types being Article (24, 44.4%), Editorial materials (20, 37.0%) and Meeting abstract (8, 14.8%). Twenty-four (44.4%) papers were published in the year of 2019, followed by 14 (25.9%) in 2020, and in addition to USA, the top 2 countries/regions of the coauthors were England (6, 11.1%), and Netherlands (5, 7.4%). It was also noticed that in addition to US FDA the top two co-authoring organizations were Brigham and Women's Hospital (7, 13.0%) and Harvard Medicine School (7, 13.0%). The top two publication categories were Pharmacology pharmacy (29, 53.7%) and Public environmental occupation health (10, 18.5%).

To be more exact, most outcomes were about the applications of RWD/RWE in different areas such as cancer, cardiovascular outcome, vaccines surveillance, medical product safety, drug prescription, precision medicine, etc. This was followed by developing different methods (including biostatistics methods) for aggregating/incorporating RWD/RWE. There were also a few papers on quality assessment/ascension, structured template, or the race/ethnicity evaluation for RWD/RWE.

#### Regulatory Guidelines

These workshops and projects involving different stakeholders were positioned to promote the issuance of the FDA's regulatory guidelines. FDA's Center for Devices and Radiological Health (CDRH) first released “Use of Real-World Evidence to Support Regulatory Decision-Making for Medical Devices Guidance for Industry and Food and Drug Administration Staff” on August 31, 2017 (19). The main content included the definition and scope of RWD/RWE, the regulatory environment and key features for medical devices. Examples generalized from the actual uses of RWE in support of regulatory decision making were provided in this guideline, including expanding indications, post-market surveillance studies, post-approval device surveillance as approval conditions, control groups, supplementary data, objective performance standards, and performance goals. Thereafter, the U.S. Department of Health and Human Services (HHS) and FDA jointly issued the “Use of Electronic Health Record Data in Clinical Investigations: Guidance for Industry” on July 18, 2018, aiming to simplify clinical research and promote the use HER data in clinical research. This guideline specified interoperability and integration techniques of HER and provided best practices for using EHR in clinical investigations.

According to the requirements of the US Congress, FDA launched “Framework for FDA's Real-World Evidence Program” on December 6, 2018 (18). In this framework, RWD was defined as “data relating to patient health status and/or the delivery of health care routinely collected from a variety of sources” and RWE as “the clinical evidence about the usage and potential benefits or risks of a medical product derived from analysis of RWD.” This framework could be used to evaluate the potential use of RWE serving as a relatively clear roadmap for how to use RWE in supporting decision-making. The main content focused on four aspects: (1) the definitions of RWD and RWE and the scope of application under the 21st Century Cure Act; (2) the use of RWD to generate RWE; (3) the RWD/RWE evaluation framework for regulatory decision making; and (4) FDA's internal and external involvement with relevant stakeholders in the development of RWE. On May 8, 2019, FDA's Center CDER and the Center for Biologics Evaluation and Research (CBER) jointly issued “Submitting Documents Using Real-World Data and Real-World Evidence to FDA for Drugs and Biologics Guidance for Industry” (20). This guidance demonstrated how companies were able to use RWD/RWE to help support their applications at the FDA, and how RWD/RWE could be used to support regulatory decision-making regarding safety and effectiveness. On January 25, 2021, CDER released the guidance documents it is planning to issue in 2021, covering 18 categories and a total of 105 new or revised guidelines.^14^ In terms of RWE, three guidelines will be developed: (1) Real-World Data: Assessing Electronic Health Records and Medical Claims Data to Support Regulatory Decision-Making for Drug and Biological Products, (2) Regulatory Considerations for the Use of Real-World Data and Real-World Evidence to Support Regulatory Decision-Making for Drugs and Biological Products, and (3) Using Registries as a Real-World Data Source for FDA Submissions. The RWD/RWE regulations and guidelines in US was summarized in Supplementary Table 1.

#### Networks and Databases

In May 2008, the FDA launched a project named the Sentinel Initiative to proactively monitor medical products on the market by leveraging the existing automated medical health data systems. During 2009 to 2014, Harvard Pilgrim Healthcare institute established the MSCC. From September 2014 to February 2016, “Mini-Sentinel” was transformed to “Sentinel System” completely. At present, the sentinel system can analyze the information of more than 300 million people and cooperate extensively with scientific research institutions, which can provide regular technical support. In early 2019, the FDA released “Five-Year Strategic Plan for the Sentinel System: 2019–2023^15^.” In this strategic plan, five aims were elaborated, and in particular one of the aims was to accelerate access to and broaden the use of RWD in evaluating effectiveness of pharmaceutical products.

FDA also participated in the establishment of some international databases with the first one being the HCV-TARGET for which FDA has been a partner since its launch in 2011. It is a cooperative academic consortium designed to inform ongoing changes in the treatment and research of hepatitis C (21). HCV-TARGET established a common research database to evaluate the use of newly approved HCV drugs in a real clinical practice setting. HCV-TARGET has registered more than 10,000 patients treated with FDA-approved HCV direct-acting antiviral drugs. The CDM project is a multiagency collaboration led by the FDA (22), aiming to capture data by combining various RWD-based data networks and simultaneously implementing them across different health care systems by mapping to a specific CDM with a consistent format and content (FHIR format). The research data could be extracted from at least four research networks: FDA's Sentinel system, Patient-Centered Outcomes Research Network (PCORNET), Informatics for Integrating Biology and the Bedside (i2b2), and Observational Medical Outcomes Partnership (OMOP). Overall, this project had established a data infrastructure to collect massive RWD to promote RWE generation.

### Outcomes

A certain number of drugs had been granted the FDA approval with applications that used RWE as part of the supporting information. These drugs could be summarized into three categories: (1) RWE for safety evaluation (pre-approval and postmarked surveillance testing); (2) efficacy evaluation (orphan drugs); and (3) new indication for already-approved drugs. For instance, in 2010, FDA announced the approval of glucosidase alpha (Lumizyme) for the treatment of infantile paroxysmal Pompe disease for patients up to 8 years of age. The registry data showed increased survival at 18 months in Lumizyme patients compared with age and disease-matched historical controls (23). In 2017, Brineura was approved for Late Infantile Neuronal Ceroid Lipofuscinosis type 2(CLN2) after FDA accepted the results from a non-randomized single-arm trial which compared with patients from an untreated natural history cohort (24). In 2018, FDA approved Lutathera (lutetium Lu 177 dotatate) by accepting an RCT with 229 patients and a single-arm, open-label study of 1,214 patients with somatostatin receptor-positive tumors (25). In 2019, Pfizer's Ibrance (Palbociclib) was approved by providing data from EHRs and post-marketing report to expand the indications to include breast cancer in men. In this case, FDA accepted the real world data from IQVIA's prescription and medical claims databases, Flatiron Health's Breast Cancer database and Pfizer's global safety database (24).

In addition to single drug for specific disease, FDA also approved TB Alliance's pretomanid tablets as part of a combination regimen with bedaquiline and linezolid for the treatment of people with a specific type of highly treatment-resistant tuberculosis (TB) of the lungs (26). There was a case of transcatheter aortic valve replacement (TAVR) device. In 2011, FDA approved first generation TAVR device for the treatment of aortic stenosis. Based on the post-market surveillance information of national device registries of TAVR, FDA approved third generation TAVR for intermediate-risk patients in 2017.^16^ It has now become a trend for many medical and health technology companies to cooperate with industry and FDA to conduct real-world evidence research based on real-world databases. For FDA, Sentinel system could also provide real world data to evaluate safety signals for safety assessments and risk management. In summary, FDA has regulated the scope and application scenarios of RWE in accordance with the requirements of US Congress. FDA has also actively cooperated with different stakeholders to improve the traditional drug development process and to bring about potential social and economic benefits.

### European Union—European Medicines Agency

#### The Aims of EMA for RWE Development

EMA is a decentralized agency of the EU, where its status is equivalent to the FDA in the US. It is responsible for the scientific evaluation, supervision, and safety monitoring of medicines in the EU. The main functions of EMA are to provide scientific drug consultants and assessments to protect human and animal health, establish European standards for human and veterinary drugs, check and follow up drugs entering the EU (27). RWE is not a new topic in the EU; it was already used to demonstrate the efficacy or safety in medicine post-authorization and rare disease where randomized clinical trials was not ethical (28). The European Parliament and European Council approved Regulation (EU) No. 1235/20101 (amending Regulation (EC) No 726/2004) and Directive 2010/84/EU2 in 2010 (amending Directive 2001/83/EC) on 15 December 2010.^17^,^18^ The national competent authorities may require the additional monitoring for specific medicinal products to marketing authorisations holders, like conducting post-authorization safety study (PASS) and post-authorization efficacy study (PAES) when there are safety risks, or the significant needs of efficacy revision.^19^ RWE plays a significant role in PASS and PAES in supporting pharmacovigilance activities (29), refining and assessing safety signals. The definition of RWD in the EU is the data relating to patient health status or the delivery of health care routinely collected from a variety of sources rather than traditional clinical trials. RWE is the information derived from analysis of RWD. In EMA, RWE is widely used in restricting and extending indications, making labeling changes, accessing benefit-risk, and the withdrawal of marketing authorization. Nearly 20% of the withdrawals in the EU are related to real world safety data.^20^

### Inputs and Activities

#### Knowledge and Technology Support

EMA delivered training curriculum on RWE, pharmacoepidemiology, methodology, and Big Data for assessors in committee assessment, where the contents were discussed at Pharmacovigilance Risk Assessment Committee (PRAC), Committee for Medicinal Products for Human Use (CHMP) and Biostatistics Working Party. Relevant training curriculums on assessment of herbal medicinal products were also developed by Committee on Herbal Medicinal Products (HMPC).^21^ In 2019, the EMA issued “Regulatory Science to 2025,” which aimed to establish a more adaptive regulatory system to encourage medical innovation (30). In this strategy, expertise to regulate product dossiers was required for EMA working group. Training curriculum of skilled analysis, collaborations with external experts from academia, recruitment on multiple disciplines (data science, biostatistics, epidemiology, advanced analytics, and AI+), and continuing education were stressed to enhance reviewers' consistent understanding of RWD source and the generation of RWE, especially from observational studies (30). A joint task force of the EMA and Heads of Medicines Agencies (HMA) was created in 2017, which mandated to make approaches to the use of “big data” in EU medicine regulatory paradigm. One of 10 priority recommendations of Phase II reports, a Methodologies Working Party (including RWD) was recommended to be established based on the existing working party (31).

#### Research and Project Funding

EMA launched framework contracts with academic and research institutions to conduct EMA-funded efficacy or safety research. Nineteen external studies based on multi-database and multinational collaboration were conducted to support EMA committees from 2010 to 2019 (32). Pharmacoepidemiologic Research on Outcomes of Therapeutics by a European Consortium (PROTECT) was a collaborative research project, which was coordinated by EMA and GlaxoSmithKline from September 2009 until June 2015. PROTECT funded by Innovative Medicines Initiative (IMI), involving 34 multinational consortiums of academics, regulators and pharmaceutical companies. The main research results related to RWE included (1) guidance for observational studies on medicines in several databases and several countries with common protocols; (2) review of good detection practices, which improved the signal detection methods in regulatory agencies and pharmaceutical companies; (3) recommendations for benefit-risk assessment methodologies and visual representations to facilitate decision-making; (4) exploring new methods to collect data directly from patients. Furthermore, EMA preformed 88 RWE in-house studies at the request of PRAC and CHMP to Committees using databases of electronic healthcare records and claims data in Europe (e.g., The THIN, IMS FR/DE, and EudraVigilance database), where a total of 88 studies started during 2013–2019.^22^ In 2020, EMA signed three contracts for observational research with academic and private partners to monitor the efficacy and safety of COVID-19 vaccines and medicines in the real world, where “COVID-19 infection and medicines in pregnancy” and “Vaccine Covid-19 monitoring readiness” project with Utrecht University, “Multicentre cohort studies on the use of medicines in COVID-19 patients” project with company IQVIA. EMA also supported the registration of post-authorization studies in the European Union electronic Register of Post-Authorisation Studies, which is one of the largest inventories of observational studies in the world.

#### Workshops and Projects

EMA organized and conducted series of workshops, meetings, and projects to explore the use of RWE throughout the medicine life cycle in the EU, and bring together various stakeholders from healthcare regulators, academia, and industry. Pharmacovigilance activities, big data in medicine regulation, evidence generation of pre-authorization and post-authorization, techniques of data characterization and discoverability, and the applications of RWE in specific drugs/therapeutics development were widely discussed (e.g., cancer drugs, orphan drug, pediatric drugs, vaccines, and advanced therapies). In addition to conducting workshops on specific topics, EMA also launched workshops to collect expert opinions on designing and drafting RWE related projects, strategies, and work plans.

Real-world data is considered as a subset of big data in EMA, so that workshops around big data have been continually organized since 2016. Workshops topics involved the potential applications, opportunities, and challenges of “big data” in medicines development and regulatory science.^23^ In 2017, HMA/EMA Joint Big Data Task Force was established, which composed of experienced medicines regulators from 14 National Competent Authorities (NCAs), EMA, and European Commission. This task force aims to present recommendations to unlock the potential of big data for medicine regulatory decision-making. Two reports and priority actions were put forward from a regulatory perspective. Pharmacovigilance Risk Assessment Committee (PRAC) of EMA adopted workshops about the strategy of risk management measures and processes to enhance the pharmacovigilance activities in the EU. Projects focus on real time monitoring of drug use patterns was developed by EMA and European Network of Centers for Pharmacoepidemiology and Pharmacovigilance (ENCePP).

EMA coordinated with multi-national medicine regulatory authorities to strengthen the safety, efficacy, and quality of medical products. Several processes and tools were developed to provide earlier access to promising medicines for unmet medical needs. EMA encouraged companies to request for Scientific Advice/Consultation throughout the life cycle of specific medicine, which were provided by the Scientific Advice Working Party of EMA. This procedure has proved to be helpful for supporting medicine marketing authorization applications and facilitating access to medicines (33). In 2017, EMA and FDA developed a plan to provide sponsors with FDA-EMA Parallel Scientific Advice to simultaneously exchange opinions with drug applicants on scientific issues during the new drug approval stage and avoid unnecessary duplication (34). Priority Medicine is an early access procedure to strengthen the development of promising new medicines, which have potentials to meet the unmet medical needs in EU (35). Adaptive Pathways is a program that enables a promising drug to be approved in a progressive approval and promotes timely access to new drugs for patients. A drug may be initially approved for a small group of patients who could get the biggest benefits, while further evidence of use may be collected over time as the supplement for the expansion of target users or indications (36). In this process, the application of RWE is indispensable, where supplementary clinical trial data and evidence about drugs in a real-world setting would be collected to support decision making, such as expanding descriptions or adding new use indications of products that have been approved. Similar early access tools include conditional marketing authorization, authorization under exceptional circumstances and accelerated assessment (37). All these processes/tools fairly promote the generation of RWE in a practical setting, which was considered as the supplement of clinical trial data in medicine evaluation.

EMA also conducted workshops and initiatives to enhance the generation of RWE from patient registries and observational studies. Patients' registries could provide post-licensing evidence of medicines or treatments of patients with particular diseases (38). The EMA Patient Registry Initiative was conducted in 2015 to facilitate use of disease registries by standard methodological approaches to support the benefit-risk evaluation of medicine.^24^ This initiative and related workshop made big progress on medicine authorization and regulatory guidance. In 2017, EMA and Drug Information Association organized a statistics forum to explore the role of observational data in assessing the benefits and risks of medicines; relevant regulatory guidance was also discussed.^25^

The Innovative Drug Initiative (IMI) is a public-private partnership between EU and European pharmaceutical industry.^26^ IMI mainly aims at improving the European drug research environment and promoting new drug development. The members including regulatory authorities, pharmaceutical companies, academia, HTA bodies, physicians, and patients. IMI supports many projects to generate data/evidence that is of direct relevance to regulatory authorities, health technology assessment (HTA) bodies and payers. There are two continuous projects focusing on generation and implementation of RWE in the EU, which are IMI GetReal (2013–2017) and IMI GetReal initiative (2018–2030 April 2021). IMI GetReal project aimed to promote the development of new methods to generate RWE and explore how to implement the collection and synthesis of RWD in medical research and healthcare decision-making in Europe. Thirty plus research publications, RWE related tools, and skills development training were delivered. GetReal projects addressed different objectives by comprising 4 work packages (WP1–4).^27^ WP1 included three parts: A Think Tank for RWE recommendations generation, a number of Task Forces for challenges solutions and a RWE Research Community for involving multiple members to provide feedback on guidelines, recommendations and reports produced by the project. WP2 focused on the long-term sustainability of GetReal Initiative on a not-for-profit basis, where WP3 addressed the overall project management and communications and WP4 for relevant ethics issues. The main tools including RWE Navigator (39), Pragmatic (40), Aggregate Data Drug Information System (41), and Sure-Real (42). In 28 April 2021, the GetReal Institute will be launched,^28^ which was formed as a non-profit multi-stakeholder organization in Netherlands. This institution was built on the success of the two previous projects. Three areas were focused: (1) reducing barriers to the secondary use of data sources for health care decision-making; (2) bridging the gap between RWE and conventional randomized controlled trial approaches, and (3) addressing the evidence needs of “downstream” decision-makers. In addition to GetReal, Recognizing Adverse Drug Reactions project 1 and 2 (IMI WEB-RADR 1 and 2) were launched in 2014 and 2018 by IMI to strengthen the power of social media for pharmacovigilance.^29^ Patients could report the medicine side effects and receive reliable information on their drugs by Med Safety mobile applications.^30^ This project could collect real-world data of medicine use for pharmacovigilance purposes (43).

### Outputs

#### Publications and Reports

From Web of Science database, the academic outputs regarding “RWD/RWE” from EMA were summarized. Twenty-one papers were generated with the top three document types being Article (12, 27%), Editorial materials (4, 19%), and meeting abstract (4, 19%). Ten (47.6%) papers were published in the year of 2020, followed by 4 (19%) in 2019 and 2017, and the top two co-authoring countries/regions were Netherlands (16, 76%), and England (15, 76%). It was also noticed that the top two co-authoring organizations were Harvard Medicine School (7, 33.3%) and US FDA (6, 28.6%). The top 2 publication categories were Pharmacology pharmacy (14, 66.7%) and Public environmental occupation health (8, 38%).

To be more exact, most outcomes were about the applications of RWD/RWE in different areas such as vaccines surveillance, medical product safety, drug prescription, Pharmacovigilance, Pharmaceuticals, and health care decision making. This was followed by developing different methods to improve the credibility of RWD/RWE via improving transparency, validity, etc. There was also one output about EMA's experience in RWD/RWE and one paper highlighting the challenges and possible solutions for Europe.

#### Regulatory Guideline

In 2019, EMA launched the OPTIMAL (Operational, TechnIcal, and MethodologicAL) framework to explore the appropriate use of valid RWE for the regulatory purpose. The challenges with the use of RWD to generate acceptable RWE in each areas (optional, technical, and methodological) could be addressed by possible solutions in EU context (32). In early 2019, the first report by HMA-EMA joint Big Data Task Force reviewed the landscape of big data and identified opportunities of big data in improving the medicine regulation (44). The final (Phase II) report was adopted by Management Board of EMA (31). In this report, practical steps that should be taken to increase the capacity of dealing with big data was determined, where 10 priority recommendations were identified, including (1) Deliver a sustainable platform (DARWIN) to access and analyze healthcare data from across the EU; (2) Establish an EU framework for data quality and representativeness; (3) Enable data discoverability; (4) Develop EU regulatory skills in big data; (5) Strengthen EU regulatory processes for big data submissions; (6) Build EU regulatory capability to analyze big data; (7) Modernize the delivery of expert advice; (8) Ensure data are managed and analyzed within a secure and ethical governance framework; (9) Collaborate with international initiatives on big data; and (10) Create an EU big data “stakeholder implementation forum.” EMA issued “Regulatory Science to 2025” strategic reflection in 2020 based on the outcomes of public consultation, workshops, and meetings, which aimed to establish a more adaptive regulatory system to encourage medical innovation. Improving the application of high-quality RWD was considered a priority to regulatory decision-making by EMA (29). Guideline on registry-based studies was drafted in May 2020, which aimed to improve the use of registry information with regulatory purpose.^31^ On 25 November 2020, European Commission issued the Pharmaceutical Strategy for Europe. Commission emphasized the digitalization and innovation in the use of RWD could improve the medicine development, authorization, and use. Pharmaceutical legislation of new methods of evidence generations and assessment would be considered by Commission.^32^ The main RWE related regulations in EU were summarized in Supplementary Table 2.

#### Networks and Databases

EMA-EUnetHTA collaboration (45) was a center founded in 2010, aiming to harness synergies between regulatory evaluation and HTA throughout the lifecycle of a pharmaceutical product. Most HTA bodies encourage the use of the existing registries of good quality to generate the RWD. This collaboration could optimize the data collection and analyze RWD (including registries) by developing standards to optimize the generation of post-licensed evidence for decision making and improve the efficiency and quality of data.^33^ European Network of Centers for Pharmacoepidemiology and Pharmacovigilance (ENCePP) was coordinated by the EMA (46), aiming to involve experts and resources in pharmacoepidemiology and pharmacovigilance across Europe and provide a platform for collaboration. The main research interests were drug safety, risk and benefit, disease epidemiology, and drug utilization. One of the key outputs of ENCePP was the establishment of a database that included numerous RWDs, such as patient registries from EU research organizations and networks (47).

EU Common Data Model (CDM) is a model to support regulatory decision-making in Europe. This program was conducted to establish HMA-EMA Joint Big Data taskforce, which aimed to describe the big data landscape from a regulatory perspective. EMA led a conference entitled “EU Common Data Model?—Why? Which? How?^34^” in 2017. The opportunities and challenges of using a common data model and what kind of guidelines should be developed for such a model were widely discussed. The similar data model included Observing Medical Outcomes Partnership (OMOP) (48) and Outpost Data Model. The establishment of “Data Analysis and Real-World Interrogation Network (DARWIN)” is the top one priority recommendation in HMA-EMA Joint Big Data Phase II report. DARWIN is an EU platform to access and analyze real world healthcare data. High-quality and robust RWE could be generated to inform regulatory decision in the EU, including support product development, medicine authorization and effects monitoring. The Big Data Steering Group was set up in February 2020 to provide suggestions to EMA Management Board and HMA on implementation of the priority recommendations. Its workplan 2020–2021 was adopted in July 2020.^35^

European Health Data and Evidence Network (EHDEN), Accelerated development of vaccine benefit-risk collaboration in Europe (ADVANCE), and Vaccine monitoring Collaboration for Europe (VAC4EU) are three RWE-related network/collaboration funded by IMI. EHDEN is launched to covert European data for 100 million individuals into the Observational Medical Outcomes Partnership common data model, which allows for the systematic analysis of disparate observational databases. ADVANCE is a public-private consortium composed of 47 organizations. It aims to deliver best evidence timely to support vaccination decision making in Europe (49). VAC4EU is the sustainability solution of the ADVANCE (ended in March 2019). It is a multi-stakeholder international association, which enables robust and timely evidence-generation on the effects of vaccines. These three initiatives will provide RWE on COVID-19 vaccines and treatment to EMA in clinical practice (50).

In order to leverage quality data for use by public authorities in November 2020, the Commission and the German Presidency of Council of the EU announced to work together to establish European Health Data Space, which is one of the priorities of the Commission 2019–2025.^36^ This initiative aims to provide a common data sharing and exchange framework across EU Member States to facilitate the use of quality health data throughout the EU. Issues on data protection rules, relevant IT systems, digital health services, and artificial intelligence in health will be clarified in following roadmap, which is under the public consultation period.

### Outcomes

Metformin is one of the most common prescribed oral anti-diabetics therapy in treatment of insulin dependent type 2 diabetes in the EU (51). CHMP reviewed the available data in previous research in real world setting to support labeling changes including a revision of the indications or contraindications. Similarly, some new drugs were approved or enlisted by applying RWE. Eculizumab is a monoclonal antibody manufactured by the company Alexion. In 2015, Alexion extended indications by providing a prospective, observational study using data from a PNH registry.^37^ Elosulfase alfa, marketed by the company BioMarin under the tradename Vimizin, was approved for conditional reimbursement in the UK, where collaboration was conducted with the MPS Society and NHS England to collect patient data in supporting the MPS IVA registry.^38^

### China—National Medical Products Administration

#### The Aims of NMPA for RWE Development

National Medical Products Administration (NMPA) is the Chinese agency for regulating medicines and medical devices (formerly the China FDA)^4^ In the past few years, NMPA has investigated how to apply RWE to the development of medicines and medical devices. Based on the main themes of the official documents released by NMPA, the aim of NMPA regarding RWE application was “development, evaluation and authorization of medicines and medical devices.” Related regulatory guidelines have provided definitions and technical supports to the development of RWE or real-world research in specific areas such as medical devices and pediatric drugs. In particular, “real-world data refers to various data related to the patient's daily health status and/or diagnosis, treatment, and health care. Not all real-world data can produce real-world evidence after analysis, and only real-world data that meets applicability criteria can be formed after proper and sufficient analysis.”^39^

### Inputs and Activities

#### Knowledge and Technology Support

As of the study being conducted, no public information was found from NMPA about knowledge and technology support for RWE (e.g., training curriculum).

#### Research and Project Funding

In April 2019, NMPA launched the China Drug Regulatory Scientific Action Plan and identified nine key research projects, including one project on RWE entitled “Methodological Research on Using Real-World Data for Clinical Evaluation of Medical Devices.” This project was led by the Department of Medical Device Supervision and Administration of NMPA, aiming to explore the use of RWD for regulation, provide solutions to accelerate the launch of innovative products, and promote reform of the medical device approval system. Then the Medical Device Regulatory Science Research Institute of Sichuan University was established and served as the first medical device regulatory scientific research base of the NMPA. From 10/Jan/2020 to 09/April/2021, there were a total of 820 studies addressing various aspects of Novel Coronavirus Pneumonia (COVID-19), which were registered at the Chinese Clinical Trials Registry at http://www.chictr.org.cn/enIndex.aspx. Amony them, a total of 17 studies were observational studies. No public information was available on whether these observational studies were supported by the NMPA or not.

#### Workshops and Projects

The 7th China Pharmacovigilance Conference was held by the National Center for Adverse Drug Reaction Monitoring on November 15, 2019. During the conference, organizers hosted a special workshop entitled “Regulatory Science and Real-World Evidence.” The aim of this workshop was to promote the use of RWE to support market-based drug safety regulatory decisions. Experts discussed the application value of RWE in medicine innovation and safety surveillance.^40^ In the same month, 2019 Real World Data Conference was held in Tianjin city. This conference was sponsored by the China Center for Food and Drug International Exchange, co-organized by the Peking University Clinical Research Institute and the Chinese Medical Doctors Association. Domestic and foreign experts from regulators, academia and industries shared their own researches or experiences on hot topics related RWD/RWE, including the background and conception, methodologies of data collection, evidence evaluation and applications.^41^

“Hainan Boao Lecheng International Medical Tourism Pilot Zone” in Hainan Province, China was established by the State Council of the People's Republic of China with nine health related preferential policies granted, including accelerated approval of medical device and medicine import registration (47). Based on the China Regulatory Scientific Action Plan for Drug Supervision, application of clinical RWD was one of the key research projects. In September 2019, the Food and Drug Administration of Hainan Province released implementation plan for pilot project of clinical RWD application in the Hainan Boao Lecheng International Medical Tourism Pilot Zone (48). A series of workshops and projects were conducted around the use of RWE in medical devices and medicine. The topics mainly focus on the feasibility potential risks and difficulties of using RWE, especially the application of RWE from the perspective of the use of medical devices and new approaches for developing medical device review and approval system were largely discussed.^42^ 2020 Real World Data Research Conference was held in Boao Lecheng on 25th September, which involved 800+ experts, researchers and regulatory stakeholders. In this conference, the main topic is the role of RWE in regulatory decision, including creating medical device supervision system with Chinese characteristics, consolidating the system foundation for the pilot work of clinical RWD application, RWD supports drug development and clinical evaluation of medical devices and how to speed up the construction of a free trade port to help RWE related research in the future.^43^ In order to support the registration and declaration of drugs and medical device products in the Hainan Pilot Zone, a team of 51 academicians and experts was introduced to guide applicants and clinical organizations to develop relevant evidence through data collection, system processing, and statistical analysis with appropriate analytical models.^44^

### Outputs

#### Publications and Reports

Different from EMA or FDA, no publication about RWD/RWE was found to be authored by researchers from the NMPA. Instead, two main national journals were found to be governed by the NMPA, China including Chinese Journal of Pharmacovigilance and China Food and Drug Administration Magazine. Fourteen relevant papers were found from Chinese Journal of Pharmacovigilance, where all except one were about the applications of RWD/RWE in different areas such as drug/medical product surveillance, drug safety evolution, Pharmacovigilance, etc. The only exception was on big data application in RWD/RWE. While nine relevant papers were found from China Food and Drug Administration Magazine, all were about the applications of RWD/RWE in different areas with the only exception being AI in RWD/RWE.

#### Regulatory Guidelines

The guidelines issued by NMPA always involved not only policy and regulatory decision-makers but also stakeholders from industry and academia. On May 29, 2019, the Center for Drug Evaluation (CDE) of NMPA released “Key Considerations in Using Real-World Evidence to Support Drug Development (Draft Version)” for public review, where comments were widely sought from May to August 2019. Afterwards, CDE of NMPA organized an expert finalization meeting and internal discussions to analyze all feedbacks and solicited opinions. “Guiding Principles of Real-World Evidence supporting Drug Development and Review (Trial)” was finally promulgated on January 7, 2020. These two regulatory guidelines gave a clear explanation about the conception of RWE in China, clarified relevant definitions of real-world research, explained the status and scope of real-world evidence, explored the evaluation principles and provided scientific guidance for industry to use RWE to support drug research and development.^45^

On December 13, 2019, the Department of Medical Device Supervision and Administration of NMPA released “Technical Guidelines for Clinical Evaluation of Medical Devices using Real-World Data (Draft Version)” to standardize the application of RWD in evaluation of medical devices, which was officially promulgated in November 2020. This guideline aimed to provide suggestions for applicants to use RWD for medical devices registration and provide technical guidance for regulatory authorities in approving relevant RWD applications.^46^ Based on the demand of “Guiding Principles of Real-World Evidence supporting Drug Development and Review,” CDE of NMPA drafted the “Guiding Principles for Real World Evidence to Support Pediatric Drug Development and Review (Draft for comments)” on May 18, 2020, which was officially promulgated on September 08, 2020.^47^ This guideline pointed out that RWE could be used as an aid to provide support for children's clinically reasonable medication evidence (e.g., new medicines for children, expansion of children's indications, and improvement of dosages for children.) to support regulatory decision-making. High quality RWD is the basis for RWE generation. For this, the CDE of NMPA organized the drafting of the “Guiding Principles for Real-World Data Used to Generate Real-World Evidence (Trial),” which was released in April 2021.^39^ The main RWE related regulations in China were summarized in Supplementary Table 3.

#### Networks and Database

With the development of RWE related activities of the NMPA, some provinces and cities in China began to explore RWE locally. Hainan Real World Data Research Institute was established in 2020, and the Food and Drug Administration of Hainan Province planned to build a real-world big data platform across the province to integrate medical data, health insurance data and drug utilization data, and to connect with other national health databases. This platform was set not only for drug registration, but also for post-market supervision and disease prevention research based on the big data and artificial intelligence environment.^48^ Hainan Key Laboratory of Real-World Data Research and Evaluation was established by NMPA in February 2021, which aimed to improve drug supervision and accelerate high-quality development of the pharmaceutical industry in China.^49^

### Outcomes

In October 2018, bevacizumab was approved for new indications by the CDE of NMPA. This approval uses retrospective real-world research, including the results of three recent Chinese retrospective real-world studies on advanced non-small cell lung cancer (52). On March 26, 2020, one glaucoma drainage device was approved with the submission of clinical RWE of racial differences. It was since approved for the surgical management of refractory glaucoma and could reduce the incidence of adverse events. On January 26, 2020, one precision laser system was approved for registration. These two cases both are medical product which was granted NMPA approval by providing clinical RWE collected from Boao Lecheng International Medical Tourism Pilot Zone.^50^

To make the comparative results more intuitive and readable, the different elements of the logic model (Input, Activities, Outputs and Outcomes) regarding the three DRAs (e.g., FDA, EMA, and NMPA) were summarized in Table 2.

**Table 2:** Logic model of RWE development in FDA, EMA, and NPMA.

**DRAs**	**INPUTS**	**ACTIVITIES**		**OUTPUTS**	**OUTCOMES**
	**What DRAs invested**	**What DRAs did**	**Who DRAs reached**	**What DRAs got**	**What DRAs achieved**
**FDA**	**Aim: “Utilizing Real World Evidence.”: supporting the approval of new indications for approved drugs and post-approval research requirements**				
	· Staff and experts · Financial · Technology · Equipment and platform. · Community · Domestic and international partners	· Conduct workshops and meetings · Provide project findings · Together stakeholders · Develop reports, curricula, resources · Facilitate access to information · Work with partners	· Industry · Academic institutions · Domestic government institutions · Overseas institutions	· Publications and reports · Regulatory guidelines · Database and data sharing platforms	· Decision-making: ∙ New drugs approved · Awareness · Knowledge · Skills · Motivations · Behavior · Health · Social · Economic
**EMA**	**Aim: Utilizing the power of big data and improving the application of high-quality RWD to support regulatory decision-making**				
	· Staff and experts · Financial · Committee · Technology · Resources and organization	· Conduct workshops and meetings · Organize initiatives · Together stakeholders · Facilitate access to information · Work with partners from different countries	· Industry · Academia · Agencies and community · Decision-makers · EU country health institutions · Overseas institutions	· Publications and reports · Centers and committees · Tools developed by activities. · Annual conference reports · Database and data sharing platforms	· Decision-making: ∙ New drugs approved and indications added. · Communications · Motivations · Behavior · Health · Social · Economic
**NMPA**	**Aim: Utilizing the power of big data and improving the application of high-quality RWD**				
	· Financial · Technology · Domestic partners	· Conduct workshops and meetings · Organize projects · Together stakeholders · Facilitate access to information · Leadership in local institutions	· Industry · Academia · Local institutions	· Publications and reports · Regulatory guidelines · Regulatory Science Action Plan	· Decision-making: ∙ New devices approved and indications added. · RWE platform · Motivations · Health · Social · Economic

## Discussion

RWE has received widespread attention and its use for regulatory decision-making has been promoted worldwide in recent years. Due to various unique health systems and institutional settings, different countries/regions have designed different roadmaps to develop RWE that suits their own situations. In this study, we summarized the definitions, scopes and developments of RWD and RWE from the official perspective of the DRAs namely FDA in US, EMA in EU, and NMPA in China.

The pharmaceutical industry is well-developed in the US. It has the largest number of innovative pharmaceutical companies and is accompanied by huge R&D investment (53). The development of RWE in the US first originated from US Congress's request to the FDA to assess potential utilization of RWE, including supporting new indications approval and post-market research. FDA actively cooperates with different stakeholders through funding, workshops, conferences, and activities to clarify how to use RWE to support regulatory decision making in the medical devices and drugs scientifically and rationally. From the activities and regulations, it can be seen that the main role of RWE in the US is to support drug approval decisions and accelerate the listing of domestic drugs. The FDA has also provided industry with guidelines on how to submit relevant RWD/RWE documents to regulatory agencies. The EU pharmaceutical industry is also a mature Industry. EU is the world's largest regional market composed of many developed countries and one of the most important mainstream pharmaceutical consumer markets (54). RWD/RWE is not a novel concept in the EU. RWE was originally used to evaluate the safety and efficacy of medicines after authorization. RWE was also widely applied in rare or orphan disease (5), in which RCTs may be unfeasible and perhaps considered unethical due to a very limited population. As the potential of big data was discovered in EU, RWE, as a subset of big data, is accepted wider for use in the entire life cycle of drugs, including accelerating approvals, and label expansions. Compared with the US and Europe, the development of the pharmaceutical industry in China is relatively backward. Although China's pharmaceutical market ranks second in the world, the number of innovative drugs only accounts for 6% of the world. As of 2020, the total number of global biopharmaceutical companies has reached 4,362, of which 76% are concentrated in Europe and the US, and the sales of European and US companies account for 93% of the global biopharmaceutical company sales.^51^ In China, one of the main applications of RWE is to evaluate the safety of imported drugs. There are corresponding guidelines and applications in drug development, pediatric drugs, medical devices, and quality standard of high-quality RWD. A special application of RWE in China is that unique traditional Chinese medicine empirical formulas could become the scope of practical evidence, including clinical research of Traditional Chinese Medicine, re-evaluation, and effectiveness analysis (55).

It is shown that there exist some subtle differences in the definitions of RWE across the FDA, EMA, and NMPA. In particular, FDA defined RWD as data relating to patient health status and/or the delivery of health care routinely collected from a variety of sources and RWE as the clinical evidence about the usage and potential benefits or risks of a medical product derived from analysis of RWD (18); from EMA perspective, RWD denoted the data relating to patient health status or the delivery of health care routinely collected from a variety of sources rather than traditional clinical trials, and RWE is the information derived from analysis of RWD (44); in NMPA, China, RWD refers to various data related to the patient's daily health status and/or diagnosis, treatment and health care, and only RWD that meets applicability criteria can be formed as RWD after proper and sufficient analysis. From the slightly different definitions of RWD in the three countries/regions, different pharmaceutical and regulatory environments can be glimpsed: (1) the scopes of RWD and RWE in US are comparatively wider than the EU and China, which is mainly due to the US's particular objective to support and accelerate approvals of new medicine; (2) the EU is comparatively conservatory in using RWE, which is primarily to address the important questions that cannot be answered in standard RCTs. However, data from patients' registry and observational study is acceptable for EMA. One persuasive reason may contribute to the potential of providing insight into post-marked safety/ efficacy issues (56), (3) the source of RWD in China is wide, however, it has relatively strict requirements on RWE. This is mainly due to the fact that RWE in China is mainly used to develop local drug R&D and review imported drugs, only the RWE available to specific disease or treatment could be scientifically accepted. Therefore, the different definitions of RWD/RWE can reflect the differences in the purpose and needs of developing RWE in these three DRAs. In addition, combined with their investment in RWE, which was summarized in the logic model. The three institutions are at different stages of RWE development. In US and EU, the development of RWE has entered a relatively mature stage, relevant regulations and pilot projects have achieved direct outputs and promote the practical applications of RWE in the fields of drugs, medical devices and treatment methods (32, 56). While RWE is in a rapid development stage in China, some relevant regulations have been issued. Projects, RWD platforms and key laboratories are being implemented and established. More inputs in staff education and direct research funding, and outputs of projects have not yet been publicly presented.

From the logic model applied in this research, although different strategies of DRAs to develop RWE could be refined, there are some common characteristic worthy of being put forward. FDA, EMA, and NMPA all engaged heavily with scholars and experts from academia and industry and facilitated cross-sector communication through workshops, conferences, and projects. Regulatory guidelines evolving around specific diseases, treatment, patient populations or technologies have been developed overtime as some of the immediate output. RWE was commonly used to support drug regulatory decisions, including providing evidence of effectiveness and safety for the registration and marketing of new drugs, providing evidence for changes in the label of a marketed drug, and providing evidence for post-marketing requirements or re-evaluation (24). RWE is also related to the overall development of regulatory science in these three countries/regions. In the US, Centers of Excellence in Regulatory Science and Innovation are the institutions that cooperated most with the FDA on RWE-related projects (57). Promoting use of high-quality real-world data (RWD) in decision-making is one of the core strategies of Regulatory Science to 2025 in EU. This strategy includes 10 priority recommendations under Big Data will be conducted by PRAC and CHMP (58). In China, RWD for Clinical Evaluation of Medical Devices is one of the key projects in the Drug Regulatory Science Action Plan. Another consensus is that high quality RWD is the basis of RWE generation (44, 56). FDA, EMA, and NMPA have established databases, data sharing platforms and structured data quality standards to ensure the production of high-quality RWD. FDA, EMA, and Health Canada have developed frameworks and programs to promote the use of high-quality RWD and to support the identification of opportunities where RWE can enhance clinical trials by overcoming clinical trial limitations (18, 59). In terms of informing regulatory-decision, EMA has cooperated with FDA and Japan regulatory partners on orphan drug supervision. Nearly a third of the orphan drug certification applications submitted to EMA in 2017 were submitted to another regulatory agencies in parallel (33). China has fewer international corporations on data sharing and REW generation. In order to improve and exploit the healthcare data potential in China, the NMPA has issued regulations to develop high-quality RWD and data establish sharing platform (60). The topics around the definition of RWD/RWE, sources of RWD, data standardization and harmonization, and generation of high quality RWD/RWE should be discussed and explored by international collaborations. In general, the entire medical and health ecosystem, including regulatory agencies, medical and health institutions, and pharmaceutical companies in various countries and regions, needs to strengthen the unified understanding of RWD and RWE, and accelerate the research and application of RWE in the ecosystem to achieve the greatest medic.

There are also specific limitations and challenges in the development of REW in these three countries/regions. For the medical data, privacy protection, data sharing and data standardization still need more technical and statistical supports. More guidance on new types of data (such as health mobile data, electronic wearable device data) and the acceptability of overseas health care data (interoperability) should be generated, and well-connected or managed registration systems should be established. Especially in EU, fragmentation heterogeneity, and lack of transparency existing in many European electronic healthcare databases. For the project cooperation plan of multiple institutions, universally accepted methodological standards should be applied to increase transparency and reliability of generated RWE (61). The development of artificial intelligence technology will also promote the development of RWE, and regulatory agencies will also face interdisciplinary challenges (62). One research implied that, although RWD informed various aspects of drug development and improved decision making, the development of RWD was largely realized well in high-income countries. More effort should be input to improve RWD utilization in a global health context (63).

The authors acknowledge the following limitations of the study: (a) this work represents a snapshot of the development, adoption, and advancement of RWE in the regulatory landscape in three countries/regions only. The findings presented in this study are not exhaustive as updates about RWE development emerges regularly, (b) to the authors' knowledge, all relevant information from the FDA, EMA and NMAP repositories was gathered but the manual data-mining process precludes absolute certainty, (c) regarding the findings about the European countries, the study primarily focused on the RWE development promoted by the EMA and actions and initiatives taken by national competent authorities in the region included in this study was limited, (d) all the results and conclusions were based on the publicly available information at the FDA, EMA, and NMPA repositories, which represents a fraction, but estimated as the most significant perspective, of the overall RWE development in the countries/regions.

## Conclusions

In conclusion, by systematically retrieving and comparing RWE related information from different sources including the DRAs' official websites and academic databases, it is shown that significant progress has all been made in the development of RWE in the US, EU, and China. Generally, various workshops and projects are organized to promote the development of RWE in medicine review and post-marketing supervision so that the corresponding regulations can be improved and implemented.

Analysis via logic model shows that the regulators of DRAs of these countries and regions have different development strategies and key areas, driven by different sets of regulatory challenges and goals unique to the corresponding situations. These differences are mainly brought by the different purposes of developing RWE corresponding to the different development stages of the pharmaceutical industry. RWE's development in the US and EU is more advanced, such as accelerating local drug review and label expansions, while RWE in China is mainly used to develop local medicine R&D and review imported medicines.

All in all, it is important to establish regulatory systems of RWE based on consensus across various sectors of pharmaceutical industry and medical development among DRAs to enhance regulatory efficiency and provide better outcomes for patients, all for better health care decisions. In addition, more detailed RWE guidance for specific areas (e.g., diseases with unresolved needs, special patient groups, technical specifications, etc.) should be prioritized according to the health needs of the people the DRA serve.

## Data Availability Statement

The original contributions presented in the study are included in the article/Supplementary Material, further inquiries can be directed to the corresponding author/s.

## Author Contributions

ML, CU, and HH conceived and designed this study. ML, SC, DY, YL, ZL, JS, JW, and HL collected the data. ML and SC drafted the manuscript. ML, CU, and HH finalized the final manuscript. All authors revised the manuscript and approved the final version submitted.

## Conflict of Interest

The authors declare that the research was conducted in the absence of any commercial or financial relationships that could be construed as a potential conflict of interest.

## Footnotes

^1^*ICH Global Meeting on E8(R1)*. Available online at: https://www.ich.org/news/summary-report-ich-global-meeting-e8r1-available-now-ich-website (accessed April 2021).

^2^*Search for FDA Guidance Documents*. Available online at: https://www.fda.gov/regulatory-information/search-fda-guidance-documents#guidancesearch (accessed April 2021).

^3^*Search Federal Register Documents Since 1994*. Available online at: https://www.federalregister.gov/ (accessed April 2021).

^4^*European Medicines Agency*. Available online at: https://www.ema.europa.eu/en (accessed April 2021).

^5^*European Commission*. Available online at: https://ec.europa.eu/info/index_en (accessed April 2021).

^6^*National Medical Product Administration*. Available online at: https://www.nmpa.gov.cn/ (accessed April 2021).

^7^*China Pharmaceutical Information*. Available online at: http://www.cpi.ac.cn (accessed April 2021).

^8^*Completed PDUFA VI Deliverables*. Available online at: http://www.fda.gov/ForIndustry/UserFees/PrescriptionDrugUserFee/ucm446608.htm (accessed April 2021).

^9^*FDA Discusses RWD, RWE With Industry, Academia*. Available online at: https://www.raps.org/news-and-articles/news-articles/2019/7/fda-discusses-rwd-rwe-with-industry-academia (accessed April 2021).

^10^*Real-World Evidence Collaborative*. Available online at: https://healthpolicy.duke.edu/projects/real-world-evidence-collaborative (accessed April 2021).

^11^*FDA-AACR Real World Evidence Workshop*. Available online at: https://www.aacr.org/professionals/policy-and-advocacy/regulatory-science-and-policy/events/fda-aacr-real-world-evidence-workshop/ (accessed April 2021).

^12^*Harnessing the Digital Revolution for Medical Device Evaluation*. Available online at: https://www.pharmacy.umaryland.edu/centers/cersievents/deviceeval/ (accessed April 2021).

^13^*FDA, Brigham and Women's Hospital Expand RCT DUPLICATE Project*. Available online at: https://www.clinicaltrialsarena.com/news/fda-rct-duplicate-project/ (accessed April 2021).

^14^*Guidance Agenda: Guidance Documents CBER is Planning to Publish During Calendar Year 2021*. Available online at: https://www.fda.gov/vaccines-blood-biologics/biologics-guidances/guidance-agenda-guidance-documents-cber-planning-publish-during-calendar-year-2021 (accessed April 2021).

^15^*Sentinel System 5-Year Strategy 2019-2023—FDA*. Available online at: https://www.sentinelinitiative.org/news-events/publications-presentations/sentinel-system-five-year-strategy-2019-2023 (accessed April 2021).

^16^*FDA Facts: Postmarket Patient Registry Ensures Access to Safe and Effective Devices*. Available online at: https://www.fda.gov/about-fda/innovation-fda/fda-facts-postmarket-patient-registry-ensures-access-safe-and-effective-devices (accessed April 2021).

^17^*Regulation (EU)No 1235/2010 of the European Parliament and of the Council*. Available online at: https://eur-lex.europa.eu/LexUriServ/LexUriServ.do?uri=OJ:L:2010:348:0001:0016:EN:PDF (accessed April 2021).

^18^*Directive 2010/84/EU of the European Parliament and of the Council*. Available online at: https://eurlex.europa.eu/LexUriServ/LexUriServ.do?uri=OJ:L:2010:348:0074:0099:EN:PDF (accessed April 2021).

^19^Amending, as regards pharmacovigilance, Directive 2001/83/EC on the Community code relating to medicinal products for human use. Available online at: https://eur-lex.europa.eu/LexUriServ/LexUriServ.do?uri=OJ:L:2010:348:0074:0099:EN:PDF (accessed April 2021).

^20^*Big Data Steering Group workshop*. Available online at: https://www.ema.europa.eu/en/documents/work-programme/workplan-hma/ema-joint-big-data-steering-group_en.pdf (accessed April 2021).

^21^*EMA ANNUAL REPORT 2019*. Available online at: https://www.ema.europa.eu/en/documents/annual-report/2019-annual-report-european-medicines-agency_en.pdf (accessed April 2021).

^22^*EU Framework for RWE Real World Evidence and Regulatory Decision Making*. Available online at: http://www.cspscanada.org/wp-content/uploads/ARLETT-Peter-Session-1-European-Medicines-Framework-for-RWE.pdf (accessed April 2021).

^23^*Identifying Opportunities for ‘Big Data' in Medicines Development and Regulatory Science*. Available online at: https://www.ema.europa.eu/en/documents/report/report-workshop-identifying-opportunities-big-data-medicines-development-regulatory-science_en.pdf (accessed April 2021).

^24^*Patient Registry Initiative—Strategy and Mandate of the Cross-Committee Task Force*. Available online at: https://www.ema.europa.eu/en/documents/other/patient-registry-initiative-strategy-mandate-cross-committee-task-force_en.pdf (accessed April 2021).

^25^*European Medicines Agency (EMA)/Drug Information Association (DIA) statistics forum: The role of observational data in assessing the benefits and risks of medicines*. Available online at: https://www.ema.europa.eu/en/events/european-medicines-agency-ema-drug-information-association-dia-statistics-forum-role-observational (accessed April 2021).

^26^*About IMI*. Available online at: https://www.imi.europa.eu/about-imi (accessed April 2021).

^27^*The Main Work Groups*. Available online at: https://www.getreal-initiative.eu/work/ (accessed April 2021).

^28^*RWE for Better Health-Care Decision-Making*. Available online at: https://www.getreal-institute.org/about-us/ (accessed April 2021).

^29^*WEB-RADR: Recognising Adverse Drug Reactions*. Available online at: https://web-radr.eu/ (accessed April 2021).

^30^*Med Safety App*. Available online at: https://web-radr.eu/mobile-apps/med-safety/ (accessed April 2021).

^31^*Guideline on Registry-Based Studies*. Available online at: https://www.ema.europa.eu/en/guideline-registry-based-studies#draft-under-public-consultation-section (accessed April 2021).

^32^*A Pharmaceutical Strategy for Europe*. Available online at: https://ec.europa.eu/health/human-use/strategy_en (accessed at April 2021).

^33^*EUnetHTA Joint Action 3 WP5 Strand B: Post-Launch Evidence Generation (PLEG) and Registries*. Available online at: https://eunethta.eu/wp-content/uploads/2020/05/EUnetHTA-PLEG_CP_01_ECFSPR_summary-report.pdf (accessed April 2021).

^34^*A Common Data Model in Europe? – Why? Which? How?* Available online at: https://www.ema.europa.eu/en/events/common-data-model-europe-why-which-how (accessed February 2021).

^35^*Big Data Steering Group*. Available online at: https://www.ema.europa.eu/en/documents/work-programme/workplan-hma/ema-joint-big-data-steering-group_en.pdf (accessed April 2021).

^36^*European Health Data Space*. Available online at: https://ec.europa.eu/health/ehealth/dataspace_en (accessed April 2021).

^37^*Soliris*. Available online at: https://www.ema.europa.eu/en/medicines/human/EPAR/soliris (accessed February 2021).

^38^*Vimizim*. Available online at: https://www.ema.europa.eu/en/medicines/human/EPAR/vimizim (accessed February 2021).

^39^*Guiding Principles for Real-World Data Used to Generate Real-World Evidence*. Available online at: http://www.ccpie.org/cn/yjxx/yphzp/webinfo/2020/08/1592161975419093.htm (accessed April 2021).

^40^*China Pharmacovigilance Conference*. Available online at: http://zgywjj.fhui.org/Page (accessed February 2021).

^41^*2019 Real World Data Research Conference*. Available online at: http://www.ccpie.org/cn/hyzl/webinfo/2019/06/1561929590858071.htm (accessed February 2021).

^42^*Real World Evidence Seminar in Boao*. Available online at: http://hifda.hainan.gov.cn/zxdt/gzdt/201901/t20190129_3095690.html (accessed February 2021).

^43^*2019 Real World Data Research Conference*. Available online at: http://www.ccpie.org/cn/hyzl/webinfo/2020/09/1601216992843088.htm (accessed February 2021).

^44^*Brief introduction of Hainan Boao Lecheng International Medical Tourism Pilot Zone*. Available online at: http://en.lecityhn.com/2020-07/01/c_505419.htm (accessed April 2021).

^45^*Guiding Principles of Real-World Evidence Supporting Drug Development and Review*. Available online at: http://www.ccpie.org/cn/yjxx/yphzp/webinfo/2020/01/1573207428041366.htm (accessed February 2021).

^46^*Technical Guidelines for Clinical Evaluation of Medical Devices using Real-World Data*. Available online at: https://www.cmde.org.cn/CL0066/20139.html (accessed February 2021).

^47^*Guiding Principles of Real-World Evidence supporting Drug Development and Review*. Available online at: http://www.ccpie.org/cn/yjxx/yphzp/webinfo/2020/09/1599938859069310.htm (accessed February 2021).

^48^Implementation of clinical real-world data application pilot in Lecheng. Available online at: http://hnrb.hinews.cn/html/2019-10/16/content_3_1.htm (accessed February 2021).

^49^*Notice of the State Food and Drug Administration on Recognizing the Second Batch of Key Laboratories*. Available online at: http://www.gov.cn/zhengce/zhengceku/2021-02/14/content_5587073.htm (accessed April 2021).

^50^*First Medical Device Product Using Domestic Real-World Data was Approved in China*. Available online at: https://www.nmpa.gov.cn/zhuanti/ypqxgg/gggzjzh/20200326212201538.html (accessed February 2021).

^51^*Analysis of the Development Status of China's Generic Drugs*. Available online at: https://www.lexology.com/library/detail.aspx?g=4066fd3f-15bc-4e3a-97b9-0b6afae82fee (accessed April 2021).
