# Supplementary material for: Integrating Real-World Evidence in the Regulatory Decision-Making Process: A Systematic Analysis of Experiences in the US, EU, and China Using a Logic Model
Source: Front Med (Lausanne). 2021 May 31;8:669509. doi: 10.3389/fmed.2021.669509 (PMC8200400; doi:10.3389/fmed.2021.669509)
Supplement: Supplementary file 1 [file Data_Sheet_1.docx]

Supplementary Material

Table S1| List of relevant RWD / RWE regulations and guidelines in US.

| **Document Title** | **Issuing Year** | **Issuing Organization / Department** | **Main content** |
| --- | --- | --- | --- |
| The 21st Century Therapy Act  (PUBLIC LAW 114-255) | 2016.12 | U.S. Congress | **SEC. 505F. UTILIZING REAL WORLD EVIDENCE**   - **Establish a program to evaluate the potential use of RWE**   1. Support the approval of a new indication for a drug approved   2. Support or satisfy post approval study requirements - **RWE defined** - **Requests FDA to develop a program to assess the potential use of real-world evidence**   1. Within 2 years: framework established, including contents and consultation      1. Contents: sources of RWE; gaps in data collection activities; standards and methodologies for collection and analysis of RWE; and priority areas, challenges, and opportunities      2. Consultation:         - Regulated industry, academia, medical professional organizations, representatives of patient advocacy organizations, consumer organizations, disease research foundations, and other interested parties         - Public-private partnership; contract, grant, or other arrangement; public workshops - **Program implementation** - **Guidance for industry**: within 5 years - **Rule of construction**:   1. Sufficient basis is needed for no specified use   2. Standards of evidences and secretary’s authority |
| Prescription Drug User Fee Act Reauthorization (PDUFA VI)  (PUBLIC LAW 112-144) | 2017.10 | U.S. Congress | - **Requests FDA to enhance use of RWE for use in regulatory decision-making**  1. Before FY 2018: complete public workshops with key stakeholders 2. Before FY 2019: initiate (or fund by contract), appropriate activities 3. Before FY 2020: publish draft guidance on how RWE can contribute to the assessment of safety and effectiveness in regulatory submissions |
| Using Real World Evidence to Support Medical Device Regulatory Decisions-A Guide for Industry and Food and Drug Administration Staff  (FDA-2016-D-2153) | 2017.8.31 | Center for Drug Evaluation and Research of FDA | - **Introduction, scopes, and background** - **Definition** of RWE in medical devices - **Regulatory context** in which RWE may be used   1. General considerations for the use of RWE   2. Application of investigational device exemption (IDE) requirements in 21 CFR 812 to the collection of RWD  1. **Characteristics of RWD** 2. Relevance 3. Reliability    - Data accrual    - Data assurance - Quality Control  - **Examples** where RWE is used   1. Expanded indications for use   2. Postmarket surveillance studies (Section 522)   3. Post-approval device surveillance as condition of approval   4. Control group   5. Supplementary data   6. Objective performance criteria and performance goals |
| FDA Real World Evidence Program Framework. | 2018.12 | FDA | - **Introduction**   1. Definitions of RWD&RWE      1. Clinical trials and observational studies covered by the RWE program   2. Scope of RWE program - **Current use of RWD for evidence generation**   1. Generating evidence regarding safety and effectiveness   2. Supporting FDA’s regulatory decisions of effectiveness   3. Trial designs using RWD to generate evidence      1. Randomized controlled trials integrated into health care systems      2. Observational studies using RWD to generate RWE - **Framework for evaluating RWD/RWE for use in regulatory decisions**  1. Using trials or studies for effectiveness decisions 2. Assessing fitness of RWD for use in regulatory decisions    1. Assessing data reliability (data accrual and data assurance) and relevance    2. Addressing gaps in RWD sources 3. Potential for study design to support effectiveness    1. Randomized designs Using RWD    2. Non-randomized, single arms trials with external RWD control.    3. Observational studies 4. Regulatory considerations for study designs    1. Use of electronic source data    2. Regulatory considerations for clinical studies generating RWE 5. Data Standards: appropriate data standards for integration and submission to FDA  - **Stakeholder engagement**   1. Internal engagement   2. External engagement |
| Submitting Documents Using Real-World Data and Real-World Evidence to FDA for Drugs and Biologics Guidance for Industry  (2019-09529  ) | 2019.5.8 | Center for Drug Evaluation and Research and Center for Biologics Evaluation and Research of FDA | - **Introduction and background** - **Example submissions using RWD and/or RWE** - **Identifying REW submitted as part of a regulatory submission**   1. Purpose of using RWE as part of the regulatory submission      1. To provide evidence in support of the effectiveness or safety for a new product approval      2. To provide evidence in support of labeling changes for an approved product      3. To be used as part of a post marketing requirement to support a regulatory decision   2. Study design using RWE      1. Randomized clinical trial      2. Single arm trial      3. Observational study      4. Other study design.   3. RWD source(s) used to generate RWE      1. Data derived from electronic health records      2. Medical claims and/or billing data      3. Product and/or disease registry data      4. Other data source that can inform on health status |

Table S2 | List of relevant RWD/RWE regulations and guidelines in European Union

| **Document Title** | **Issuing Year** | **Issuing Organization / Department** | **Main content** |
| --- | --- | --- | --- |
| Regulation (EU) No. 1235/20101 (amending Regulation (EC) No 726/2004) laying down Community procedures for the authorization and supervision of medicinal products for human and veterinary use and establishing a European Medicines Agency, and Regulation (EC) No 1394/2007 on advanced therapy medicinal products. And Directive 2010/84/EU2 in 2010 (amending Directive 2001/83/EC) on 15 December 2010. | 2010.12.15 | The European Parliament and European Council | - Competent authorities may also require additional monitoring for specific medicinal products that are **subject to the obligation to conduct a post-authorisation safety study** or to conditions or restrictions with regard to the safe and effective use of the medicinal product. - The Commission should be empowered to adopt supplementary measures laying down the situations in which **post-authorisation efficacy studies may be required**. It is of particular importance that the Commission carry out appropriate consultations during its preparatory work, including at expert level. - **Post-authorisation safety study**: Any study relating to an authorised medicinal product conducted with the aim of identifying, characterising or quantifying a safety hazard, confirming the safety profile of the medicinal product, or of measuring the effectiveness of risk management measures. - In addition to the provisions laid down in Article 19, **a marketing authorisation for a medicinal product may be granted** subject to one or more of the following conditions:  1. to take certain measures for ensuring the safe use of the medicinal product to be included in the risk management system. 2. to **conduct post-authorisation safety studies**; 3. to comply with obligations on the recording or reporting of suspected adverse reactions which are stricter than those referred to in Title IX; 4. any other conditions or restrictions with regard to the safe and effective use of the medicinal product; 5. the existence of an adequate pharmacovigilance system; 6. to conduct **post-authorisation efficacy studies** where concerns relating to some aspects of the efficacy of the medicinal product are identified and can be resolved only after the medicinal product has been marketed.  - The Commission should therefore be empowered to impose on the **marketing authorisation holder the obligation to conduct post-authorisation studies on safety and on efficacy**. - After the granting of a marketing authorisation, the Agency may impose an obligation on the marketing authorisation holder:   1. to **conduct a post-authorisation safety study** if there are concerns about the risks of an authorised medicinal product.   2. to conduct a **post-authorisation efficacy study** when the understanding of the disease or the clinical methodology indicate that previous efficacy evaluations might have to be revised significantly |
| HMA-EMA Joint Big Data Taskforce Summary report | 2019.02.13 | HMA, EMA | - **Observational data subgroup recommendations** (Electronic health records)  1. Sustainable mechanisms for combining healthcare data across Europe should be implemented.  - Evaluation criteria: The speed of RWE generation across multiple datasets. - **Spontaneous ADR subgroup recommendation**s   1. Invest in methods to link pharmacovigilance data sources with other real world clinical and nonclinical data sources. - **Social media and M-Health data subgroup recommendations** (m Health)   1. Support effective vigilance practices using state of the art m-Health technology      - Reinforcing Actions: Continue to develop apps for directly gathering data from patients on adverse events and encourage their wider use in real world and study settings |
| HMA-EMA Joint Big Data Taskforce Phase II report: “Evolving Data-Driven Regulation” | 2020.01.20 | HMA, EMA | - **Big Data** includes **real world data** such as electronic health records, registry data and claims data, pooled clinical trials data, datasets from spontaneously reported suspected adverse drug reaction reports, and genomics, proteomics and metabolomics datasets. - **10 priority recommendations**  1. **Deliver a sustainable platform to access and analyse healthcare data from across the EU (Data Analysis and Real World Interrogation Network -DARWIN)** 2. Establish an EU framework for data quality and representativeness 3. Enable data discoverability 4. Develop EU network skills in Big Data 5. Strengthen EU network processes for Big Data submissions 6. Build EU network capability to analyse Big Data 7. Modernise the delivery of expert advice 8. Ensure data are managed and analysed within a secure and ethical governance framework 9. Collaborate with international initiatives on Big Data 10. Create an EU Big Data “stakeholder implementation forum” |
| Regulatory Science Strategy to 2025 | 2020.03.31 | EMA | - Goal 1: Catalysing the integration of science and technology - in medicines development - Goal 2: Driving collaborative evidence generation-improving the scientific quality of evaluations   1. Optimise capabilities in modelling, simulation and extrapolation      - **Deploy advances in RWD**, modelling, simulation and extrapolation to benefit special populations particularly neglected patient populations - Goal 3: Advancing patient-centred access to medicines in partnership with healthcare systems  1. **Promote use of high-quality real-world data (RWD) in decision-making**    - - The actions in this Regulatory Science Strategy relating to RWD are included within the 10 actions listed under Big Data.        - Conduct a **pilot of using rapid analytics of real-world data** (including electronic health records) to support decision-making at the PRAC and CHMP        - Review of the utility of **using electronic health records for detecting drug safety issues** (including drug interactions)      - Mapping of **good examples of use of RWD** in different phases of drug development to develop guidance on such use.    1. Develop network competence and specialist collaborations to engage with big data       - **Data Analysis Real World Interrogation Network-DARWIN**       - **Modernise the delivery of expert advice**. Build on the existing working party structure to establish a Methodologies Working Party that encompasses biostatistics, modelling and simulation, extrapolation, pharmacokinetics, **real world data**, epidemiology and advanced analytics, and establish an Omics Working Party that builds on and reinforces the existing pharmacogenomics group.  - Goal 4. Addressing emerging health threats and availability/ therapeutic challenges - Goal 5. Enabling and leveraging research and innovation in regulatory science |
| Guideline on registry-based studies | 2020.09.24 | EMA | - **Introduction, scope and objective** - **Methods and processes**   1. Use of registry-based studies for evidence generation   2. Differences between a registry-based study and a patient registry   3. Planning a registry-based study   4. Study protocol   5. Study population   6. Data collection   7. Data quality management   8. Data analysis   9. Data reporting - **Legal basis and regulatory requirements** |
| Pharmaceutical Strategy for Europe | 2020.11.25 | European Commission | - Supporting a competitive and innovative European pharmaceutical industry  1. Enabling innovation and digital transformation    - The Commission will propose to revise the pharmaceutical legislation to consider how to make best use of digital transformation. This includes new methods of evidence generation and assessment, such as analysis of big and real world data to support the development, authorisation and use of medicines. |

Table S3 | List of relevant RWD / RWE regulations and guidelines in China

| **Document Title** | **Issuing Year** | **Issuing Organization / Department** | **Main content** |
| --- | --- | --- | --- |
| Guiding Principles of Real-World Evidence supporting Drug Development and Review (Trial) | 2020.01.07 | Center for Drug Evaluation of NMPA | - Relevant definitions of RWS - Source and applicability of RWD  1. Hospital information system 2. Medical claim system 3. Patients registry 4. China ADR Sentinel Surveillance Alliance 5. Population-based cohort study of a natural experiment and specific disease cohort database 6. Omics database 7. Death registration database 8. Outcome data of patient report 9. Data from the mobile device 10. Other data sources  - Data Standard:   1. Predictable   2. Consistent - Data Suitability   1. Correlation   2. reliability      - Completeness      - Accuracy      - Transparency      - quality assurance - RWE supports drug regulatory decisions  1. Provide evidence of effectiveness and safety for new drug registration 2. Provide evidence for label changes for approved drugs 3. Provide evidence for post-marketing requirements or re-evaluations 4. Explore new approaches for clinical research and development of well-known prescriptions/ formulas from Experience of the TCM practitioner 5. Guide clinical research design 6. Locate target groups  - Basic design for real-world research  1. Practical clinical trials (PCTs) 2. Single-arm trial using RWE as external control 3. Observational research  - Evaluation of RWE  1. Clinical issues supported by RWE 2. From RWD to RWE  - Communicate with review agencies |
| Technical Guidance for Using RWE to Support R&D and Regulatory Review of Pediatric Drugs (Trial) | 2020.09.01 | Center for Drug Evaluation of NMPA | - The difference and integration of real-world research and traditional RCT - Common scenarios of real-world research used in the development of pediatric drug in China  1. Post-marketing clinical safety and effectiveness study of new ingredient drugs approved for children 2. Drugs that have been approved for use in adults and children in other countries, at the same time, these drugs have been approved for use in adults in China: declare for use in Chinese children by data extrapolation strategies 3. Application of off-label drug data to support the expansion of indications to children (only for drugs have approved in China) 4. Rare disease and others  - Cases |
| Technical Guideline on Real World Data (RWD) Used in Medical Device Clinical Evaluation (Trial Implementation) | 2020.11.26 | Department of Medical Device Supervision and Administration of NMPA | - RWD and RWE - Strengths and limitations of real-world research - Common RWD sources and classifications (includes, but is not limited to)  1. Data generated from the process of providing and paying for health and medical services, such as HER data, medical insurance data, health registries, etc. 2. Based on the research purpose when the database was established, a unified data standard and data collection model were established, and data resources formed and established in routine clinical practice, such as device registration data, etc.  - RWD quality evaluation  1. Representative 2. Completeness 3. Accuracy 4. Authenticity 5. Consistency 6. Repeatability  - Common types of real-world research designs and statistical analysis methods  1. Common types of real-world research designs    - Pragmatic Clinical Trial    - Observational research    - Others 2. Statistical analysis    - Data set definition, analysis principles and strategies, missing data processing, analysis indicators and analysis methods, subgroup or stratification analysis, sensitivity analysis, supplementary analysis and result reporting, etc.  - Scenarios where RWE can be considered for clinical evaluation of medical devices  1. Provide evidence in clinical evaluation of the same type of device 2. Support product registration as a supplement to existing evidence 3. The RWD generated in the domestic licensed use of imported devices in urgent clinical need can be used to support product registration as a supplement to the existing evidence 4. External control for single-group trial 5. Provide clinical data for single-arm objective performance criteria 6. Support the modification of the scope of application, indications, and contraindications 7. Support to modify the clinical value of the product in the instructions 8. Support post-market research of conditionally approved products 9. Used for long-term safety and/or effectiveness evaluation of medical devices such as implants have a higher risk 10. Clinical evaluation of medical devices used for the treatment of rare diseases in the whole life cycle, speeding up the process of marketing, and meeting the needs of patients  - Post-marketing monitoring |
| Guiding Principles for Real-World Data Used to Generate Real-World Evidence (Draft for Comment) | 2021.04.15 | Center for Drug Evaluation of NMPA | - RWD sources - Applicability Evaluation of RWD  1. Applicability evaluation of source data 2. Applicability evaluation of governance data (relevance and reliability)  - Governance of RWD  1. Personal information protection and data security processing 2. Data extraction 3. Data cleaning 4. Data conversion 5. Data transmission and storage 6. Data quality control 7. General data model 8. Real-world data governance plan  - Compliance, security, and quality management system of RWD  1. Data compliance 2. Data security management 3. Quality management system  - Communication with regulatory agencies |
